# Association between oral anticoagulants and osteoporosis: Real-world data mining using a multi-methodological approach

**DOI:** 10.7150/ijms.39523

**Published:** 2020-02-04

**Authors:** Satoshi Yokoyama, Shoko Ieda, Mirai Nagano, Chihiro Nakagawa, Makoto Iwase, Kouichi Hosomi, Mitsutaka Takada

**Affiliations:** 1 Division of Clinical Drug Informatics, School of Pharmacy, Kindai University, 3-4-1 Kowakae, Higashi-osaka, Osaka 577-8502, Japan; 2 Department of Pharmacy, Kindai University Hospital, 377-2 Ohno-higashi, Osaka-Sayama, Osaka 589-8511, Japan

**Keywords:** warfarin, direct oral anticoagulant, osteoporosis, disproportionality analysis, sequence symmetry analysis, data mining

## Abstract

**Introduction**: Warfarin and direct oral anticoagulants (DOACs) have been widely used in antithrombotic therapy. Although warfarin use has been suspected to be associated with osteoporosis risk, several studies have shown otherwise. Conversely, a few reports have found an association between DOACs and osteoporosis. This study therefore clarifies the association between oral anticoagulants and osteoporosis by analyzing real-world data using different methodologies, algorithms, and databases.

**Methods**: Real-world data from the US Food and Drug Administration Adverse Event Reporting System (FAERS; 2004-2016) and Japanese administrative claims database (2005-2017; JMDC Inc., Tokyo) were used. Reporting odds ratio (ROR) and information component (IC) were calculated through disproportionality analysis (DPA) using reports recorded in the FAERS. Sequence symmetry analysis (SSA) was employed to calculate the adjusted sequence ratio (SR) using the JMDC Claims Database. For the adjusted SR and ROR, a significant signal was detected when the lower limit of the two-sided 95% confidence interval (CI) was more than 1. For the IC, a significant signal was detected when the lower limit of the 95% CI was more than 0.

**Results**: DPA for warfarin found significant signals for osteoporosis in ROR (1.43, 95% CI: 1.32-1.54) and IC (0.50, 95% CI: 0.39-0.61). SSA showed a significant association between warfarin use and osteoporosis or bisphosphonate use. Moreover, a significant association was observed in males and females, albeit only for warfarin.

**Conclusion**: Multi-methodological data mining revealed that warfarin use, not DOACs, is significantly associated with osteoporosis regardless of sex difference.

## Introduction

Osteoporosis is a serious health problem given that it leads to osteoporotic fractures, which cause significant decline in activities of daily living and quality of life. To make matters worse, osteoporotic fractures are becoming more frequent given the aging world population 1. Several risk factors for osteoporotic fractures have been identified, including being female, low bone mineral density, and previous fractures 2. Moreover, several drugs, such as glucocorticoids, have been reported to increase osteoporosis risk. The usage of the oral anticoagulant warfarin, a vitamin K antagonist, has also been suspected as a risk factor for osteoporosis. One study reported an association between warfarin use and low bone mineral density 3. In retrospective cohort studies, long-term exposure to warfarin was associated with higher fracture risk compared with non-exposure 4, 5. On the other hand, a prospective observational study revealed that warfarin use did not decrease bone mineral density and was not associated with fracture risk among elderly women 6. Furthermore, recent studies have revealed that the use of vitamin K antagonists seem to neither increase fracture risk nor reduce bone mineral density 7, 8. Hence, the effect of warfarin on osteoporosis remains controversial. Historically, warfarin has been the mainstay of antithrombotic therapy. However, the clinical use of direct oral anticoagulant (DOAC) therapy has considerably expanded 9. The advantages of DOACs include a fixed-dose regimen, absence of drug monitoring, and few drug-drug interactions 10. With regard to the relationship between DOACs and osteoporosis, several animal studies have implied that DOACs may have lower adverse effects on bone health than warfarin 11-13. However, little is known regarding the influence of DOACs on osteoporosis among humans.

Recently, real-world data, including claims and spontaneous adverse event report databases, have dramatically accumulated with the progression of information technology. Pharmacovigilance and pharmacoepidemiological studies using such databases have been critical for monitoring the safety of newly marketed medications 14. Various analytical methods have been developed to identify unexpected associations between drugs and adverse events. Among them, disproportionality analysis (DPA) and sequence symmetry analysis (SSA) have been used as complementary tools in pharmacovigilance 15, 16. DPA evaluates spontaneous adverse drug event report databases, whereas SSA evaluates insurance claims and prescription databases, both of which have been frequently used to predict potential association between drugs and their adverse events. Moreover, DPA and SSA are computationally expeditious approaches that employ simple algorithms, making them useful tools for pharmacovigilance. In addition, the combined use of DPA and SSA can enhance signal detection given that SSA is able to detect additional true-positive signals that are not detected by DPA algorithms alone 17. This study analyzed real-world data to clarify the association between oral anticoagulants and osteoporosis.

## Methods

### Analysis of the FAERS database

#### Data source

This study used data from the US Food and Drug Administration Adverse Event Reporting System (FAERS), the largest worldwide database for spontaneous self-reports of adverse drug events that are freely available to the public. The FAERS comprises seven datasets: DEMO, DRUG, REAC, OUTC, RPSR, THER, and INDI. To analyze this database, the aforementioned datasets were linked. The present study included data from the first quarter of 2004 to the end of 2016 with a total of 8,867,135 reports. Duplicate reports were identified through the CASE number and excluded, leaving 7,343,647 reports for analyses.

#### Identifying oral anticoagulants and osteoporosis

Arbitrary drug names, including trade names and abbreviations, were mapped into unified generic names via text mining using the Martindale website (https://www.medicinescomplete.com/mc/login.htm). Oral anticoagulants, including warfarin, dabigatran, rivaroxaban, apixaban, and edoxaban, were identified by linking this archive with the FAERS database. All records that included oral anticoagulants in DRUG files were selected, after which relevant reactions from REAC files were identified.

Adverse events in the FAERS database were coded and classified using the Medical Dictionary for Regulatory Activities (MedDRA® version 21.1) preferred terms, which are grouped in accordance with defined medical conditions of the area of interest. Spontaneously reported adverse events related to osteoporosis were identified using the preferred terms in the Standardized MedDRA® Queries (SMQ) “20000178: Osteoporosis/osteopenia.” SMQs are validated and maintained by the Maintenance and Support Services Organization and updated with each version of MedDRA®. The SMQ “20000178: Osteoporosis/osteopenia” contained 10 preferred terms (10049470: Bone density decreased, 10056809: Bone formation decreased, 10065687: Bone loss, 10064269: Bone marrow edema syndrome, 10049088: Osteopenia, 10031282: Osteoporosis, 10031285: Osteoporosis postmenopausal, 10031290: Osteoporotic fracture, 10038642: Bone resorption increased, and 10039984: Senile osteoporosis).

#### Data mining

DPA was originally developed to determine associations between the use of certain targeted drugs and potential adverse events. Accordingly, it utilizes reporting odds ratio (ROR) and information component (IC) to detect spontaneous report signals 18, 19. ROR and IC at a 95% confidence interval (CI) were calculated in accordance with methods previously described 20. Briefly, signal scores were calculated using a case/non-case method. Reports containing the event of interest were defined as cases, whereas all other reports were identified as non-cases. For the ROR, a significant signal is detected if the lower limit of the two-sided 95% CI was more than 1. For the IC, a significant signal is detected if the lower limit of the 95% CI was more than 0. In the current study, two methods were used to detect significant signals, and the potential association between oral anticoagulant use and osteoporosis was determined when the two indices met the criteria outlined above.

### SSA using JMDC Claims Database

#### Data source

The JMDC Claims Database is a large and chronologically organized claims database (JMDC Inc., Japan) that uses standardized disease classifications and anonymous record linkage 21. This database (January 2005-March 2018) includes approximately 5.5 million insured individuals in Japan (approximately 4.4% of the population) and mainly comprises company employees and their family members. This database provides information on beneficiaries, including encrypted personal identifiers, age, sex, International Statistical Classification of Diseases and Related Health Problems 10th Revision (ICD-10), and procedure and diagnostic codes as well as the name, dose, and number of days that the prescribed and/or dispensed drugs were supplied. All drugs were coded in accordance with the Anatomical Therapeutic Chemical (ATC) Classification of the European Pharmaceutical Market Research Association. An encrypted personal identifier was used to link claims data from different hospitals, clinics, and pharmacies.

#### Rationale for SSA

SSA, which carries high specificity for detecting adverse events using claims data 22, 23, was performed to evaluate the association between oral anticoagulant use and osteoporosis or bisphosphonate use. Adjusted sequence ratios (SRs) were calculated with reference to a previous report 20. Briefly, SSA evaluates the asymmetry in the distribution of an incident event before and after the initiation of a specific treatment. Asymmetry may indicate an association between a specific treatment of interest and outcome 24. Crude SR is defined as the ratio of the number of newly diagnosed osteoporosis cases after an oral anticoagulant treatment to that before initiation. In addition, SRs were adjusted for temporal trends in oral anticoagulant use and events using the method proposed by Hallas 25. The probability that oral anticoagulants were prescribed first, in the absence of any causal relationship, can be estimated by the so-called null-effect SR generated by the proposed model, which may be interpreted as a reference value for the SR. Therefore, the null-effect SR is the expected SR in the absence of any causal association after accounting for incidence trends. By dividing the crude SR by the null-effect SR, the adjusted SR corrected for temporal trends is obtained. In the present study, the lower limit of the 95% CI of an adjusted SR > 1.0 indicated a significant association between oral anticoagulant exposure and incidence of osteoporosis.

#### Data mining

In the JMDC Claims Database, outcomes were defined by either osteoporosis diagnosis or bisphosphonate use. All incident users of oral anticoagulants and all newly diagnosed osteoporosis cases or bisphosphonate users from January 2005 to March 2018 were identified. Incidence was defined as the first prescription of oral anticoagulants (ATC code: B01AA03 for warfarin, B01AE07 for dabigatran, B01AF01 for rivaroxaban, B01AF02 for apixaban, and B01AF03 for edoxaban). Target osteoporosis diagnosis was defined in accordance with ICD-10 codes M80 and M81. Bisphosphonate use was defined in accordance with ATC code M05BA for minodronate, M05BA01 for etidronate, M05BA04 for alendronate, M05BA06 for ibandronate, and B05BA07 for risedronate. To exclude persistent oral anticoagulant users, the analysis was restricted to those whose first prescription was administered in July 2005 or later (after a run-in period of 6 months). Likewise, analysis was restricted to osteoporosis cases or bisphosphonate users who were identified in July 2005 or later. Waiting time distribution analysis 26 was performed to ensure that the analysis was restricted to incident users of oral anticoagulants and newly diagnosed osteoporosis cases or bisphosphonate users. An identical run-in period was also applied to patients enrolled in the cohort after June 2005. Incident users were identified by excluding those who received their first oral anticoagulant before July 2005, whereas new osteoporosis cases or bisphosphonate users were identified by excluding those whose first osteoporosis diagnosis or bisphosphonate use was before July 2005. Those who had initiated a new oral anticoagulant treatment and whose first osteoporosis diagnosis or bisphosphonate use was within 3, 6, 12, 18, and 24 months (intervals) of treatment initiation were identified. Patients who had received their first oral anticoagulant treatment and whose first osteoporosis diagnosis or bisphosphonate use were within the same month were not included in the determination of the SR. The 95% CI for the adjusted SR was calculated using a method for determining the exact CIs for binomial distributions 27.

## Results

### DPA of the association between oral anticoagulants and osteoporosis in the FAERS database

A total of 24,772 reports for osteoporosis were identified in the FAERS database. Demographic data are shown in Table S1. Age, sex ratio, and concomitant use of prednisolone were comparable between warfarin and DOACs. There were 138,354 drug-reaction pairs for warfarin and 660 osteoporosis cases. The association between oral anticoagulants and osteoporosis based on the FAERS database is shown in Table 1. The analysis of warfarin therapy found significant signals for osteoporosis in ROR and IC.

A total of 152,709, 103,470, 50,077, 78,317, and 24,550 drug-reaction pairs for DOACs, oral direct factor Xa inhibitors, dabigatran, rivaroxaban, and apixaban were found, respectively. After analyzing the aforenoted DOACs, no significant signals in ROR and IC were found. DPA could not be performed for edoxaban because no case could be found in the FAERS database.

### SSA of the association between oral anticoagulant use and osteoporosis in the JMDC Claims Database

A total of 1,639,157 claims pertaining to osteoporosis, 122,196 patients with osteoporosis, and 71,494 incident users (males: 14,471, females: 57,023) who were initially diagnosed with osteoporosis were identified during the study period. Moreover, a total of 325,479 claims pertaining to warfarin, 17,675 warfarin users, and 7,078 incident users (males: 4,882, females: 2,196) who received their first warfarin treatment were identified. The characteristics of the study population, as well as those using other oral anticoagulants in the JMDC Claims Database, are summarized in Table S2. Table S3 outlines the characteristics of the study population with regard to outcomes. The number of osteoporosis cases was approximately four times higher among females than males. Moreover, the number of oral anticoagulant users was higher among males than females.

The SSA results for the association between oral anticoagulant use and osteoporosis diagnosis are presented in Table 2. Accordingly, warfarin use was significantly associated with osteoporosis diagnosis at intervals of 3, 6, 12, and 18 months. Although the adjusted SR was over 1.0 at the 24-month interval, it was not statistically significant. No significant association between DOAC use and osteoporosis diagnosis was observed at any interval. Furthermore, neither oral direct factor Xa inhibitors nor any of the four DOACs was significantly associated with osteoporosis diagnosis.

Bisphosphonate use was also investigated as a proxy for osteoporosis. Accordingly, 468,614 claims pertaining to bisphosphonate use, 33,575 bisphosphonate users, and 18,586 incident users (males: 3,759, females: 14,827) who received their first bisphosphonate were identified during the study period. The SSA results of the association between warfarin use and bisphosphonate use are shown in Table 3. At all intervals, warfarin use was significantly associated with bisphosphonate use. However, no significant signals were observed between other oral anticoagulants and bisphosphonate use.

### Sex difference in the association between warfarin use and osteoporosis

Females have less bone tissue and lose bone faster than males because of the changes that happen with menopause. As such, sex differences in the association between warfarin use and osteoporosis were further investigated. DPA revealed a significant association between warfarin use and osteoporosis in males (ROR: 1.75 [95% CI: 1.50-2.04], IC: 0.44 [0.55-0.99]) and females (ROR: 1.48 [1.35-1.62], IC: 0.55 [0.42-0.68]). In the JMDC Claims Database, significant signals between warfarin use and osteoporosis at the 6-month interval and between warfarin use and bisphosphonate use at the 3-month interval were observed among males (Table 4). Furthermore, significant signals between warfarin use and osteoporosis at the 3- and 12-month intervals and between warfarin use and bisphosphonate use at all intervals were detected in females. In other intervals, adjusted SRs were all over 1.0 but were not statistically significant.

## Discussion

The present data-mining study investigated whether oral anticoagulant therapy carries osteoporosis risk by analyzing several real-world data. Accordingly, signals, including ROR, IC, and adjusted SR, indicated a significant association between warfarin use and osteoporosis in every database (Table 5), whereas no significant signals were not observed for DOACs.

Osteoporosis is closely related to vitamin K levels considering that three typical vitamin K-dependent proteins, namely, osteocalcin, matrix Gla protein, and growth arrest-specific protein 6, play key functions in maintaining bone strength 28. Vitamin K, which is associated with bone mineral density 29, is an essential cofactor required for the gamma-carboxylation of these proteins. Given that warfarin inhibits gamma-carboxylation, which in turn controls a function of these proteins, the long-term use of warfarin has been suggested to be associated with osteoporosis. Moreover, dabigatran and other DOACs, which are competitive reversible antagonists of thrombin and activated factor Xa, respectively 30, have been considered to have limited influence on bone mineral density.

Our results are strongly suggestive of osteoporosis risk with warfarin use given that the DPA and SSA signals indicated a significant association between warfarin use and osteoporosis. Furthermore, the association between DOACs and osteoporosis had not been observed. One recent study found no cases of osteoporosis among their cohort of DOAC users 31, and several studies corroborate the notion that replacing warfarin with DOACs improves bone markers 32. Furthermore, a meta-analysis of randomized controlled trials showed that DOACs carry a lower risk for osteoporotic fracture compared with warfarin 33. Accordingly, all aforementioned findings are consistent with those presented herein.

Generally, females have been more susceptible to osteoporosis than males. Considering that warfarin use was associated with osteoporosis among males and females in the present study, warfarin was suggested to be significantly associated with osteoporosis regardless of sex difference.

Our analysis employed multi-methodological approaches using different methods, algorithms, and databases to acquire consistent findings from independent analyses. The DPA of spontaneous adverse drug-reaction report databases can be used to identify signals of disproportionate reporting. Moreover, DPA facilitates the identification of previously unknown, albeit clinically important, associations while providing useful suggestions to guide clinical decision making 34. By using DPA, the present study analyzed the FAERS, one of the biggest databases currently accessible to the public, subsequently detecting signals of bleeding, one of the well-known adverse events of oral anticoagulant therapy 35. This result indicates the usefulness of DPA in detecting adverse events of oral anticoagulants in the FAERS database. The present study utilized the ROR 36, given by the frequentist approach, and IC 19, given by a Bayesian confidence propagation neural network, as data-mining algorithms for the quantitative detection of signals. Considering that no individual algorithm is able to adequately detect signals, some studies use more than one algorithm. Thus, the concurrent use of several algorithms is considered essential. To strengthen DPA findings, different algorithms and databases were used for further analysis. Accordingly, SSA, the advantages of which include its efficiency in computation, moderate sensitivity but high specificity, and robustness toward time-constant confounding factors 16, was performed using the JMDC Claims Database. Outcomes were defined as osteoporosis diagnosis and bisphosphonate use. Bisphosphonates, which are potent antiresorptive agents, are the most commonly used drugs for osteoporosis 37. Consequently, significant signals were detected through DPA and SSA.

Nevertheless, each method has several limitations 16, 34. DPA and SSA using real-world data raise the possibility that the reported event was not caused by the drug given the limitations in the quality control of real-world data. Thus, not every adverse event or medication error associated with a drug is reported, and the database may contain missing data and frequent misspellings of drug names. The number of signals calculated using DPA does not provide a robust indication of signal strength. Given that control populations are not included in spontaneous reporting systems, disproportionality-based signals indicate an increased risk of adverse event reporting and not the risk of adverse events. SSA may be affected by prescription trends over time, which may possibly lead to a biased effect estimate 25. We prevented this bias by calculating null-effect SRs 24. Channeling bias 38 should also be taken into account when interpreting results from the SSA of a tested medication for which a therapeutic alternative exists given the presence of several cases switching among oral anticoagulants. In addition, DPA is affected by channeling bias. The concomitant use of corticosteroids might affect our results. Corticosteroid is associated with a dose-related increase in osteoporosis risk. The percentage of concomitant use of prednisolone that was one of the most frequently used corticosteroids in the warfarin group was comparable to that in the DOACs group (Table S1). However, the possibility of the effect of corticosteroids on our results cannot be completely denied. Individuals included in the JMDC Claims Database were selected from beneficiaries covered by the employees' health insurance system. Considering that most beneficiaries are working adults or their family members, the proportion of elderly patients aged 65 years or older remained low. One previous epidemiological study in Japan showed that the estimated annual incidence of osteoporosis among females was approximately four times more than that among males 39. Given that incident female users were approximately four times more than males in the JMDC Claims Database, the database was considered to be reflective of the Japanese population diversity. Owing to potential limitations, careful attention must to be given to the interpretation of detected signals in real-world data, and our findings certainly need further clinical investigation. Nonetheless, our results implicated that the combination of DPA and SSA strengthens the robustness of signals, and significant signals derived from a multi-methodological approach are suggested to be more reliable than those derived from other approaches.

The results of this data-mining study, which used different methodologies, algorithms, and large-scale real-world data, strongly suggest an association between warfarin use and osteoporosis. The methods of the study could be proposed in the context of signal detection for hypothesis generation, not testing the risk of adverse events. The potential association between drugs and their adverse events derived from data-mining studies 40,41 is disproved by cohort studies 42,43. It is necessary to exercise caution when interpreting the results derived from DPA and SSA and to verify the risk of osteoporosis with oral anticoagulants by performing further pharmacoepidemiological studies.

## Supplementary Material

Supplementary tables.

## Figures and Tables

**Table 1:** Disproportionality analysis of the association between oral anticoagulants and osteoporosis based on FAERS database (2004-2016)

Oral anticoagulant	Cases(n)	Non-cases(n)	ROR	95% CI		IC	95% CI	
				Lower	Upper		Lower	Upper
**Warfarin**	660	137,694	1.43*	1.32	1.54	0.50*	0.39	0.61
**DOACs**	124	152,585	0.24	0.20	0.28	-0.25	-2.30	-1.79
**Oral direct factor Xa inhibitors**	94	103,376	0.27	0.22	0.33	-1.88	-2.17	-0.59
**Dabigatran**	30	50,047	0.18	0.12	0.25	-2.45	-2.96	-1.95
**Rivaroxaban**	70	78,247	0.26	0.21	0.33	-1.90	-2.24	-1.56
**Apixaban**	25	24,525	0.30	0.20	0.44	-1.69	-2.24	-1.13
**Edoxaban**	0	1,117	**-**	**-**	**-**	**-**	**-**	**-**

FAERS: FDA Adverse Event Reporting System, ROR: reporting odds ratio, IC: information component, CI: confidence interval. Cases: number of reports of osteoporosis, Non-cases: all reports of adverse drug reactions other than osteoporosis. DOACs: dabigatran, rivaroxaban, apixaban, and edoxaban. Oral direct factor Xa inhibitors: rivaroxaban, apixaban, and edoxaban. *: significant signal, -: not applicable.

**Table 2:** The association between oral anticoagulants use and osteoporosis diagnosis in JMDC Claims Database (January 2005 to March 2018)

Oral anticoagulant	Incident users (n)	Concomitant with osteoporosis (n)	Simultaneous start (n)	Intervals (month)	Number of patients diagnosed of osteoporosis (n)		Crude SR	Null-effect SR	Adjusted SR	95% CI	
					Last	First				Lower	Upper
**Warfarin**	7,078	787	76	3	112	72	1.56	1.03	1.51*	1.12	2.07
				6	153	101	1.51	1.05	1.45*	1.12	1.88
				12	223	154	1.45	1.09	1.33*	1.08	1.65
				18	274	194	1.41	1.13	1.25*	1.04	1.51
				24	301	218	1.38	1.17	1.18	0.99	1.41
**DOACs**	13,509	1366	104	3	108	188	0.57	0.98	0.59	0.46	0.75
				6	190	271	0.70	0.96	0.73	0.60	0.88
				12	280	385	0.73	0.93	0.78	0.67	0.91
				18	340	472	0.72	0.90	0.80	0.69	0.92
				24	386	541	0.71	0.88	0.81	0.71	0.93
**Oral direct factor Xa inhibitors**	12,230	1304	102	3	103	183	0.56	0.98	0.58	0.45	0.74
				6	178	257	0.69	0.96	0.73	0.60	0.88
				12	263	370	0.71	0.92	0.77	0.66	0.91
				18	323	459	0.70	0.88	0.80	0.69	0.92
				24	361	526	0.69	0.84	0.81	0.71	0.93
**Dabigatran**	2,312	117	3	3	9	9	1.00	1.01	0.99	0.35	2.80
				6	17	18	0.94	1.03	0.92	0.45	1.89
				12	25	27	0.93	1.07	0.87	0.48	1.55
				18	28	32	0.88	1.12	0.78	0.45	1.34
				24	38	36	1.06	1.19	0.89	0.55	1.45
**Rivaroxaban**	4,384	264	9	3	17	22	0.77	1.00	0.77	0.38	1.52
				6	26	37	0.70	1.00	0.70	0.41	1.19
				12	50	58	0.86	1.00	0.86	0.58	1.29
				18	69	82	0.84	0.99	0.85	0.61	1.19
				24	80	95	0.84	0.97	0.87	0.64	1.18
**Apixaban**	3,532	248	7	3	13	16	0.81	0.99	0.82	0.37	1.83
				6	27	34	0.79	0.97	0.82	0.48	1.40
				12	44	60	0.73	0.95	0.77	0.51	1.16
				18	64	80	0.80	0.93	0.86	0.61	1.21
				24	76	94	0.81	0.90	0.90	0.66	1.23
**Edoxaban**	5,483	876	89	3	77	152	0.51	0.94	0.54	0.40	0.71
				6	132	203	0.65	0.90	0.72	0.58	0.90
				12	183	279	0.66	0.83	0.79	0.66	0.96
				18	209	327	0.64	0.76	0.85	0.71	1.01
				24	224	375	0.60	0.69	0.86	0.73	1.02

Last: number of patients treated with oral anticoagulant followed by diagnosis of osteoporosis. Fast: number of patients diagnosed of osteoporosis followed by oral anticoagulant treatment. SR: sequence ratio, CI: confidence interval. DOACs: dabigatran, rivaroxaban, apixaban, and edoxaban. Oral direct factor Xa inhibitors: rivaroxaban, apixaban, and edoxaban. Osteoporosis was defined as M80 and M81 in ICD-10 code. Number of incident user with osteoporosis: 71,494. *: significant signal.

**Table 3:** The association between oral anticoagulant use and bisphosphonate use in JMDC Claims Database (January 2005 to March 2018)

Oral anticoagulant	Incident users (n)	Concomitant with bisphosphonate (n)	Simultaneous start (n)	Intervals (month)	Number of patients with bisphosphonate use (n)		Crude SR	Null-effect SR	Adjusted SR	95% CI	
					Last	First				Lower	Upper
**Warfarin**	7,078	322	60	3	54	20	2.70	1.03	2.63*	1.55	4.64
				6	65	31	2.10	1.04	2.01*	1.29	3.19
				12	90	58	1.55	1.08	1.43*	1.02	2.03
				18	108	68	1.59	1.12	1.42*	1.04	1.95
				24	122	72	1.69	1.16	1.46*	1.08	1.98
**DOACs**	13,509	438	36	3	41	40	1.03	0.98	1.04	0.66	1.66
				6	70	65	1.08	0.97	1.11	0.78	1.58
				12	105	117	0.90	0.94	0.96	0.73	1.26
				18	118	139	0.85	0.91	0.93	0.73	1.20
				24	130	163	0.80	0.88	0.91	0.72	1.15
**Oral direct factor Xa inhibitors**	12,230	417	36	3	43	38	1.13	0.98	1.16	0.73	1.84
				6	68	58	1.17	0.96	1.22	0.85	1.76
				12	101	110	0.92	0.92	1.00	0.75	1.32
				18	113	132	0.86	0.89	0.97	0.75	1.25
				24	123	155	0.79	0.85	0.94	0.73	1.19
**Dabigatran**	2,312	40	1	3	1	4	0.25	1.02	0.25	0.01	2.49
				6	5	9	0.56	1.03	0.54	0.14	1.79
				12	11	14	0.79	1.07	0.74	0.30	1.75
				18	13	15	0.87	1.11	0.78	0.34	1.75
				24	15	16	0.94	1.17	0.80	0.37	1.73
**Rivaroxaban**	4,384	90	6	3	7	6	1.17	1.00	1.16	0.34	4.19
				6	12	16	0.75	1.00	0.75	0.32	1.68
				12	24	25	0.96	1.00	0.96	0.53	1.76
				18	27	30	0.90	0.99	0.91	0.52	1.59
				24	28	32	0.88	0.97	0.90	0.52	1.55
**Apixaban**	3,532	82	6	3	7	7	1.00	0.99	1.01	0.30	3.38
				6	9	11	0.82	0.98	0.84	0.31	2.23
				12	16	21	0.76	0.95	0.80	0.39	1.61
				18	19	26	0.73	0.93	0.78	0.41	1.47
				24	21	33	0.64	0.90	0.71	0.39	1.26
**Edoxaban**	5,483	273	27	3	30	28	1.07	0.95	1.13	0.65	1.97
				6	49	37	1.32	0.91	1.46	0.93	2.30
				12	64	74	0.86	0.84	1.04	0.73	1.47
				18	70	89	0.79	0.77	1.03	0.74	1.42
				24	77	106	0.73	0.70	1.03	0.76	1.40

Last: number of patients treated with warfarin followed by bisphosphonate. Fast: number of patients treated with bisphosphonate followed by warfarin. SR: sequence ratio, CI: confidence interval. DOACs: dabigatran, rivaroxaban, apixaban, and edoxaban. Oral direct factor Xa inhibitors: rivaroxaban, apixaban, and edoxaban. Bisphosphonates: minodronate, etidronate, alendronate, ibandronate, and risedronate. Number of incident user with bisphosphonate use: 18,586. *: significant signal.

**Table 4:** The association between warfarin use and osteoporosis diagnosis or bisphosphonate use based on sex difference

Sex	Incident warfarin users (n)	Osteoporosis diagnosis		Bisphosphonate use		Intervals (month)	Number of patients (n)		Crude SR	Null- effect SR	Adjusted SR	95% CI		
		Concomitant with osteoporosis(n)	Simultaneous start (n)	Concomitant with bisphosphonate (n)	Simultaneous start (n)									
							Last	First				Lower	Upper	
Male	4,882	348	46			3	53	36	1.47	1.03	1.42	0.92	2.24	
						6	76	43	1.77	1.05	1.68*	1.14	2.50	
						12	101	69	1.46	1.10	1.33	0.97	1.83	
						18	120	82	1.46	1.15	1.27	0.95	1.70	
						24	131	90	1.46	1.20	1.21	0.92	1.60	
				130	32	3	24	10	2.40	1.03	2.33*	1.07	5.46	
						6	25	12	2.08	1.05	1.99	0.96	4.34	
						12	37	27	1.37	1.09	1.26	0.74	2.14	
						18	41	29	1.41	1.14	1.24	0.75	2.07	
						24	47	30	1.57	1.19	1.32	0.82	2.16	
Female	2,196	439	30			3	59	36	1.64	1.03	1.59*	1.03	2.48	
						6	77	58	1.33	1.05	1.27	0.89	1.81	
						12	122	85	1.44	1.08	1.33*	1.00	1.77	
						18	154	112	1.38	1.12	1.23	0.96	1.58	
						24	170	128	1.33	1.16	1.15	0.91	1.45	
				192	28	3	30	10	3.00	1.03	2.90*	1.38	6.66	
						6	40	19	2.11	1.05	2.01*	1.14	3.67	
						12	53	31	1.71	1.08	1.58*	1.00	2.55	
						18	67	39	1.72	1.11	1.54*	1.02	2.35	
						24	75	42	1.79	1.15	1.55*	1.05	2.32	

Last: number of patients treated with oral anticoagulant followed by outcome of interest. Fast: number of patients with outcome of interest followed by oral anticoagulant treatment. SR: sequence ratio, CI: confidence interval. Osteoporosis was defined as M80 and M81 in ICD-10 code. Bisphosphonates: minodronate, etidronate, alendronate, ibandronate, and risedronate. Number of male incident user of osteoporosis and bisphosphonate use were 14,471 and 3,759, respectively. Number of female incident user of osteoporosis and bisphosphonate use were 57,023 and 14,827, respectively. *: significant signal.

**Table 5:** Summary of the results of DPA and SSA

Database and analysis			Warfarin	DOACs	Oral direct factor Xa inhibitors	Dabigatran	Rivaroxaban	Apixaban	Edoxaban
**FAERS**									
	DPA	ROR	▲	-	-	-	-	-	-
		IC	▲	-	-	-	-	-	-
**JMDC Claims Database**									
	SSA	Outcome							
		Osteoporosis diagnosis	▲	-	-	-	-	-	-
		Bisphosphonate use	▲	-	-	-	-	-	-

FAERS: FDA Adverse Event Reporting System. DPA: disproportionality analysis. ROR: reporting odds ratio. IC: information component. SSA: sequence symmetry analysis. DOAC: direct oral anti coagulants including dabigatran, rivaroxaban, apixaban, and edoxaban. Oral direct factor Xa inhibitors: rivaroxaban, apixaban, and edoxaban. Osteoporosis defined by ATC code M80 and M81. Bisphosphonates: minodronate, etidronate, alendronate, ibandronate, and risedronate. ▲: significant signal. -: not detected or not applicable

## Abbreviations

ATC: Anatomical Therapeutic Chemical
CI: confidence interval
DOAC: direct oral anticoagulant
DPA: disproportionality analysis
FAERS: Food and Drug Administration Adverse Event Reporting System
IC: information component
ICD-10: International Statistical Classification of Diseases and Related Health Problems 10th revision
MedDRA: Medical Dictionary for Regulatory Activities
ROR: reporting odds ratio
SMQ: Standardised MedDRA Queries
SR: sequence ratio
SSA: sequence symmetry analysis

## References

[1] Cummings SR, Melton LJ (2002). Epidemiology and outcomes of osteoporotic fractures. Lancet.

[2] Sambrook P, Cooper C (2006). Osteoporosis. Lancet.

[3] Philip WJ, Martin JC, Richardson JM, Reid DM, Webster J, Douglas AS (1995). Decreased axial and peripheral bone density in patients taking long-term warfarin. QJM.

[4] Gage BF, Birman-Deych E, Radford MJ, Nilasena DS, Binder EF (2006). Risk of osteoporotic fracture in elderly patients taking warfarin: results from the National Registry of Atrial Fibrillation 2. Arch Intern Med.

[5] Caraballo PJ, Heit JA, Atkinson EJ, Silverstein MD, O'Fallon WM, Castro MR (1999). Long-term use of oral anticoagulants and the risk of fracture. Arch Intern Med.

[6] Jamal SA, Browner WS, Bauer DC, Cummings SR (1998). Warfarin use and risk for osteoporosis in elderly women. Study of Osteoporotic Fractures Research Group. Ann Intern Med.

[7] Veronese N, Bano G, Bertozzo G, Granziera S, Solmi M, Manzato E (2015). Vitamin K antagonists' use and fracture risk: results from a systematic review and meta-analysis. J Thromb Haemost.

[8] Fiordellisi W, White K, Schweizer M (2019). A Systematic Review and Meta-analysis of the Association Between Vitamin K Antagonist Use and Fracture. J Gen Intern Med.

[9] Huisman MV, Rothman KJ, Paquette M, Teutsch C, Diener HC, Dubner SJ (2017). The Changing Landscape for Stroke Prevention in AF: Findings From the GLORIA-AF Registry Phase 2. J Am Coll Cardiol.

[10] January CT, Wann LS, Alpert JS, Calkins H, Cigarroa JE, Cleveland JC Jr (2014). 2014 AHA/ACC/HRS guideline for the management of patients with atrial fibrillation: a report of the American College of Cardiology/American Heart Association Task Force on practice guidelines and the Heart Rhythm Society. Circulation.

[11] Kluter T, Weuster M, Bruggemann S, Menzdorf L, Fitschen-Oestern S, Steubesand N (2015). Rivaroxaban does not impair fracture healing in a rat femur fracture model: an experimental study. BMC Musculoskelet Disord.

[12] Fusaro M, Dalle Carbonare L, Dusso A, Arcidiacono MV, Valenti MT, Aghi A (2015). Differential effects of dabigatran and warfarin on bone volume and structure in rats with normal renal function. PLoS One.

[13] Morishima Y, Kamisato C, Honda Y, Furugohri T, Shibano T (2013). The effects of warfarin and edoxaban, an oral direct factor Xa inhibitor, on gammacarboxylated (Gla-osteocalcin) and undercarboxylated osteocalcin (uc-osteocalcin) in rats. Thromb Res.

[14] Hall GC, Sauer B, Bourke A, Brown JS, Reynolds MW, LoCasale R (2012). Guidelines for good database selection and use in pharmacoepidemiology research. Pharmacoepidemiol Drug Saf.

[15] Wilson AM, Thabane L, Holbrook A (2004). Application of data mining techniques in pharmacovigilance. Br J Clin Pharmacol.

[16] Lai EC-C, Pratt N, Hsieh C-Y, Lin S-J, Pottegård A, Roughead EE (2017). Sequence symmetry analysis in pharmacovigilance and pharmacoepidemiologic studies. Eur J Epidemiol.

[17] Wahab IA, Pratt NL, Kalisch LM, Roughead EE (2013). Sequence Symmetry Analysis and Disproportionality Analyses: What Percentage of Adverse Drug Reaction do they Signal?.

[18] Rothman KJ, Lanes S, Sacks ST (2004). The reporting odds ratio and its advantages over the proportional reporting ratio. Pharmacoepidemiol Drug Saf.

[19] Bate A, Lindquist M, Edwards IR, Olsson S, Orre R, Lansner A (1998). A Bayesian neural network method for adverse drug reaction signal generation. Eur J Clin Pharmacol.

[20] Fujimoto M, Higuchi T, Hosomi K, Takada M (2015). Association between statin use and cancer: data mining of a spontaneous reporting database and a claims database. Int J Med Sci.

[21] Kimura S, Sato T, Ikeda S, Noda M, Nakayama T (2010). Development of a database of health insurance claims: standardization of disease classifications and anonymous record linkage. J Epidemiol.

[22] Pratt N, Chan EW, Choi NK, Kimura M, Kimura T, Kubota K (2015). Prescription sequence symmetry analysis: assessing risk, temporality, and consistency for adverse drug reactions across datasets in five countries. Pharmacoepidemiol Drug Saf.

[23] Wahab IA, Pratt NL, Wiese MD, Kalisch LM, Roughead EE (2013). The validity of sequence symmetry analysis (SSA) for adverse drug reaction signal detection. Pharmacoepidemiol Drug Saf.

[24] Tsiropoulos I, Andersen M, Hallas J (2009). Adverse events with use of antiepileptic drugs: a prescription and event symmetry analysis. Pharmacoepidemiol Drug Saf.

[25] Hallas J (1996). Evidence of depression provoked by cardiovascular medication: a prescription sequence symmetry analysis. Epidemiology.

[26] Hallas J, Gaist D, Bjerrum L (1997). The waiting time distribution as a graphical approach to epidemiologic measures of drug utilization. Epidemiology.

[27] Morris JA, Gardner MJ (1988). Calculating confidence intervals for relative risks (odds ratios) and standardised ratios and rates. Br Med J (Clin Res Ed).

[28] Wen L, Chen J, Duan L, Li S (2018). Vitamin Kdependent proteins involved in bone and cardiovascular health (Review). Mol Med Rep.

[29] Pearson DA (2007). Bone health and osteoporosis: the role of vitamin K and potential antagonism by anticoagulants. Nutr Clin Pract.

[30] Ahrens I, Lip GY, Peter K (2010). New oral anticoagulant drugs in cardiovascular disease. Thromb Haemost.

[31] Treceno-Lobato C, Jimenez-Serrania MI, Martinez-Garcia R, Corzo-Delibes F, Martin Arias LH (2019). New Anticoagulant Agents: Incidence of Adverse Drug Reactions and New Signals Thereof. Semin Thromb Hemost.

[32] Namba S, Yamaoka-Tojo M, Kakizaki R, Nemoto T, Fujiyoshi K, Hashikata T (2017). Effects on bone metabolism markers and arterial stiffness by switching to rivaroxaban from warfarin in patients with atrial fibrillation. Heart Vessels.

[33] Gu ZC, Zhou LY, Shen L, Zhang C, Pu J, Lin HW (2018). Non-vitamin K antagonist oral anticoagulants vs. warfarin at risk of fractures: a systematic review and meta-analysis of randomized controlled trials. Front Pharmacol.

[34] Sakaeda T, Tamon A, Kadoyama K, Okuno Y (2013). Data mining of the public version of the FDA Adverse Event Reporting System. Int J Med Sci.

[35] Alshammari TM, Ata SI, Mahmoud MA, Alhawassi TM, Aljadhey HS (2018). Signals of bleeding among direct-acting oral anticoagulant users compared to those among warfarin users: analyses of the post-marketing FDA Adverse Event Reporting System (FAERS) database, 2010-2015. Ther Clin Risk Manag.

[36] van Puijenbroek EP, Bate A, Leufkens HG, Lindquist M, Orre R, Egberts AC (2002). A comparison of measures of disproportionality for signal detection in spontaneous reporting systems for adverse drug reactions. Pharmacoepidemiol Drug Saf.

[37] Khosla S, Bilezikian JP, Dempster DW, Lewiecki EM, Miller PD, Neer RM (2012). Benefits and risks of bisphosphonate therapy for osteoporosis. J Clin Endocrinol Metab.

[38] Lobo FS, Wagner S, Gross CR, Schommer JC (2006). Addressing the issue of channeling bias in observational studies with propensity scores analysis. Res Social Adm Pharm.

[39] Orimo H, Nakamura T, Hosoi T, Iki M, Uenishi K, Endo N (2012). Japanese 2011 guidelines for prevention and treatment of osteoporosis-executive summary. Arch Osteoporos.

[40] Raschi E, Poluzzi E, Koci A, Salvo F, Pariente A, Biselli M (2015). Liver injury with novel oral anticoagulants: assessing post-marketing reports in the US Food and Drug Administration adverse event reporting system. Br J Clin Pharmacol.

[41] Maura G, Billionnet C, Coste J, Weill A, Neumann A, Pariente A (2018). Non-bleeding adverse events with the use of direct oral anticoagulants: a sequence symmetry analysis. Drug Saf.

[42] Alonso A, MacLehose RF, Chen LY, Bengtson LG, Chamberlain AM, Norby FL (2017). Prospective study of oral anticoagulants and risk of liver injury in patients with atrial fibrillation. Heart.

[43] Douros A, Azoulay L, Yin H, Suissa S, Renoux C (2018). Non-Vitamin K antagonist oral anticoagulants and risk of serious liver injury. J Am Coll Cardiol.

