# Supplementary material for: Association between oral anticoagulants and osteoporosis: Real-world data mining using a multi-methodological approach
Source: Int J Med Sci. 2020 Feb 4;17(4):471–9. doi: 10.7150/ijms.39523 (PMC7053309; doi:10.7150/ijms.39523)
Supplement: Supplementary file 1 — Supplementary tables. [file ijmsv17p0471s1.pdf]

Table S1. Characteristics of osteoporosis cases in warfarin and DOACs in FAERS database (2004-2016)

Variables	Warfarin		DOACs	
	n = 660	(%)	n = 124	(%)
Age				
Data available	506		90	
mean ±S.D.	64.0 ± 13.5		70.7 ± 12.0	
Sex				
Data available	638		119	
Male	172	(27.0)	31	(26.1)
Concomitant use				
Prednisolone	27	(4.0)	5	(4.0)
Number of concomitant drugs [median]	6		7	

DOAC: direct oral anticoagulant
FAERS: FDA Adverse Event Reporting System
S.D.: standard deviation

Table S2. Baseline characteristics of the study population with the use of oral anticoagulants in JMDC Claims Database (January 2005 to March 2018)

	Warfarin user		DOACs user											
			Total		Oral directfactor Xa inhibitors		Dabigatran		Rivaroxaban		Apixaban		Edoxaban	
Claims, n	335,262		176,392		133,964		48,605		66,965		39,553		27,626	
Users, n	17,675		17,437		14,776		3,765		5,736		4,220		6,038	
Incident users, n	7,078		13,509		12,230		2,312		4,384		3,532		5,483	
Age, n(%)														
<20	367	(5.2)	46	(0.3)	43	(0.4)	4	(0.2)	6	(0.1)	5	(0.1)	34	(0.6)
20-39	732	(10.3)	681	(5.0)	603	(4.9)	102	(4.4)	187	(4.3)	141	(4.0)	306	(5.6)
40-59	3,405	(48.1)	6,475	(48.0)	5,798	(47.4)	1,140	(49.3)	2,092	(47.7)	1,526	(43.2)	2,679	(48.9)
60-75	2,574	(36.4)	6,307	(46.7)	5,786	(47.3)	1,066	(46.1)	2,099	(47.9)	1,860	(52.7)	2,464	(44.9)
Median (range)	56	(0–75)	59	(1–75)	59	(7–75)	58	(1–75)	59	(15–75)	60	(7–75)	58	(13–75)
Male, n(%)	4,882	(69.0)	9,528	(70.5)	8,472	(69.3)	1,907	(82.5)	3,532	(80.6)	2,772	(78.5)	3,057	(55.8)

DOACs: dabigatran, rivaroxaban, apixaban, and edoxaban
Oral direct factor Xa inhibitors: rivaroxaban, apixaban, and edoxaban

Table S3. Characteristics of the study population of outcomes in JMDC Claims Database (January 2005 to March 2018)

	Osteoporosis diagnosis		Bisphosphonate use	
Claims, n	1,704,762		474,229	
Users, n	122,196		33,575	
Incident users, n	71,494		18,586	
Age, n(%)				
<20	1,584	(2.2)	215	(1.2)
20-39	4,786	(6.7)	1,447	(7.8)
40-59	35,584	(49.8)	6,944	(37.4)
60-75	29,540	(41.3)	9,980	(53.6)
Median (range)	57	(0–75)	60	(2–75)
Male, n(%)	14,471	(20.2)	3,759	(20.2)

Osteoporosis was defined as M80 and M81 in ICD-10 code.

Bisphosphonates: minodronate, etidronate, alendronate, ibandronate, and risedronate
